# Twin Pregnancies—More to Be Done

**DOI:** 10.3390/jcm11216358

**Published:** 2022-10-27

**Authors:** Matan Anteby, Liran Hiersch

**Affiliations:** 1 Lis Maternity Hospital, Sourasky Medical Center, Tel Aviv 6423906, Israel; 2 Sackler Faculty of Medicine, Tel Aviv University, Tel Aviv 4R73+8Q, Israel

Over the past few decades, we have been experiencing an increase in the incidence of multiple gestations, mostly due to the widespread use of assisted reproduction technologies. Multiple gestation encompasses 2–3% of all pregnancies, most of which are twin pregnancies [1]. However, when compared to singletons, twin gestations are associated with substantially higher rates of neonatal mortality and morbidity, mainly owing to the higher incidence of preterm birth (PTB) and deviant fetal growth [2,3]. The vast amount of complications and special character of a twin pregnancy incite numerous pregnancy-related challenges to the caregiver. Some of these challenges entail management controversies yet to be settled.

Twin pregnancies pose a significantly high risk for preterm birth. Approximately half of twins are born preterm (<37 weeks gestation), and 10% to 15% are born before the 34th week of gestation [4]. In spite of advancements in understanding the different mechanisms of PTB, a meaningful reduction in the rate of PTB has yet to be achieved, and controversies remain regarding the optimal treatment for the prevention of PTB [5,6]. Whereas progesterone usage is backed by strong evidence in selected singleton pregnancies, this is not the case with unselected twin pregnancies, and therefore specific indications for its use are needed. The same goes for cervical pessary, which was not found to be effective in large randomized trials involving singleton or twin gestations [5]. Finally, there is no defined consensus on the indications for cervical cerclage placement in twin pregnancies. This is probably due to the scant evidence regarding the efficacy of cerclage, the exact cervical length threshold that warrants the consideration of ultrasound-indicated cerclage or the criteria for physical examination-indicated cerclage in twin pregnancies [7,8,9,10]. Thus, while it is important to individualize the management of twin pregnancies with other risk factors of preterm birth, the need for ongoing research in this field is crucial.

Another major obstetric controversy related to twin pregnancies is the delayed fetal growth observed in the third trimester. Whether this phenomenon should be considered as a normal physiologic characteristic of twins or a pathologic process that warrants special attention is currently unclear and has implications on the chart that should be used for the assessment of fetal growth and on the definition of fetal growth restriction [2,11,12].

Finally, there are complications that are specific to monochorionic placentation, which are associated with high risk of perinatal morbidity and mortality as compared to dichorionic twin gestations. Much progress was made over the last few decades in the management of those complications, mainly laser ablation of placental anastomoses. However, there is still much to achieve in tailoring the optimal treatment to each patient.

In summary, twin pregnancies present unique challenges, and the optimal management of most of them is still unclear. Thus, it is important to continue with the conduction of clinical trials as well as basic-science research for the improvement of care and long-term outcomes of mothers and babies.
